# Learning what and how to describe: longitudinal development of content selection and strategy application in audio description training

**DOI:** 10.3389/fpsyg.2026.1771684

**Published:** 2026-07-01

**Authors:** Yuxin Zou, Deyan Zou, Hanyu Wang

**Affiliations:** Dalian University of Foreign Languages, Dalian, China

**Keywords:** audio description, competence development, content selection, longitudinal analysis, strategy application, student audio describers

## Abstract

Audio description (AD) is an accessibility service that conveys visual information through verbal language for visually impaired audiences. This study examines the competence development of student audio describers by tracking their progress across two critical dimensions: content selection and translation strategy application. Drawing on data from a one-semester Audiovisual Translation course, the research analyzes the performance of 26 students through diachronic and phased comparisons, complemented by questionnaire and interview data. The findings reveal distinct developmental patterns in each competence area. Content selection competence demonstrated generally linear improvement, with a notable acceleration occurring after the fourth phase, followed by divergent trajectories among different student groups. In contrast, the application of translation strategies evolved in a more fluctuating and intuitive manner, developing independently rather than in parallel with content selection competence. This study provides empirical insights into AD training and contributes to pedagogical understanding of competence development in audiovisual translation education.

## 1 Introduction

Audio description (AD) originated in the United States in the 1970s to assist visually impaired individuals in accessing audiovisual content (Snyder, 2005). Despite decades of development, its implementation remains uneven worldwide. In China, where an estimated 17 million visually impaired people live, access to quality AD is still limited due to low public awareness, insufficient legal frameworks, and the lack of widely adopted professional standards (Yan and Luo, 2019).

In this context, training qualified audio describers is essential. While previous research has examined AD products and audience reception (Abu Tair et al., 2023; Asimakoulas, 2024) or explored course design and competence definitions (Mazur and Chmiel, 2021; Jankowska, 2019), far less attention has been paid to how describers acquire and refine their skills, particularly in underdeveloped contexts such as China.

To address this gap, this study investigates the competence development of student audio describers in a Chinese university, focusing on two core dimensions, content selection and translation strategy application, through structured classroom training and project-based learning.

## 2 Literature review

### 2.1 Audio description training

Drawing on Jakobson’s (1959) foundational framework, AD is recognized for its intersemiotic nature, involving the transposition of visual and auditory semiotic content into verbal language. Within translation studies, AD has frequently been situated within the broader field of audiovisual translation (AVT) and discussed in this intersemiotic context (Braun and Starr, 2021; Reviers, 2018). While AVT courses have been offered since the late 1980s, standalone AD courses remain relatively rare and are often embedded as modules within broader AVT curricula (Talaván et al., 2023). In recent years, however, the field has witnessed increased efforts toward professionalization and standardization, exemplified by the publication of training guidelines and EU-funded initiatives yet (ADLAB PRO IO2, 2017; Fryer, 2016). Research on AD training can broadly be grouped into two pedagogical strands: curriculum design and specialized applications.

The dominant strand focuses on course structure and competence exploration. Large-scale surveys have examined differences between academic and non-academic training contexts in terms of content, activities, and targeted competences (Mazur and Chmiel, 2021), while other studies have sought to define the core competences required of professional audio describers, particularly at postgraduate level (Mendoza Domínguez and Matamala, 2020). Experiential learning approaches, such as museum-based AD internships, have also been proposed as a means of enhancing practical relevance and employability (Luque Colmenero and Soler Gallego, 2019). Together, this line of research promotes competence-oriented training models aligned with professional practice.

Beyond traditional accessibility training, AD has also been adopted as a pedagogical tool in adjacent fields. In foreign language education, AD tasks have been shown to support the development of fluency, prosody, and mediating competence in both learners’ first and foreign languages (Navarrete, 2018; Pintado Gutiérrez and Torralba, 2022). Similarly, in interpreter education, AD practice has been found to foster expressive competence, cognitive coordination, and social inclusion awareness (Yan and Luo, 2023; Zhan and Moratto, 2024). In parallel, scholars have argued for the integration of critical accessibility perspectives to promote ethical awareness and learner agency in training contexts (Greco, 2020).

### 2.2 Competence development in audio description practice and pedagogy

Research on translator competence development has long focused on instructional methods, learning processes, and assessment frameworks within formal training settings (Galán-Mañas and Hurtado Albir, 2015; Albir et al., 2020). Extending this line of inquiry to media accessibility, scholars generally agree that AD competence is a multifaceted construct encompassing linguistic, technical, ethical, and user-oriented dimensions.

AD-specific competence research has primarily addressed conceptualization and pedagogical modeling. Early studies identified gaps in AD training literature and proposed comprehensive instructional frameworks, often informed by situated learning theory and projects such as ADLAB PRO (Chmiel et al., 2019). Other work has reviewed competence definitions and reflected on their pedagogical application (Matamala and Orero, 2007), or documented classroom practices to illustrate how linguistic precision, audience awareness, and creativity emerge through training (Jankowska, 2019). Empirical studies have further reinforced the importance of aligning educational design with professional standards by surveying practitioners and companies to identify key competences and professional expectations (ADLAB PRO IO2, 2017; Mendoza and Matamala, 2019), including in cross-cultural contexts (Tor-Carroggio and Casas-Tost, 2020).

Given that AD fundamentally involves decisions about what, how, when, and how much to describe, yet existing guidelines consistently stress that descriptions should not interfere with the original soundtrack (Remael et al., 2015). Among these dimensions, the what and how are widely regarded as the most cognitively and technically demanding and have therefore attracted particular scholarly attention.

The what dimension refers to content selection, namely the identification of narratively salient and meaningful visual elements for verbalization. Decisions in this dimension are shaped by narrative relevance and visual prominence, and are also influenced by language-specific foregrounding patterns, as evidenced by comparative corpus research (Wang et al., 2024). The how dimension concerns strategy application, that is, the linguistic rendering of selected visual information into coherent and stylistically appropriate verbal output. Research in this area has identified a range of AD-specific strategies, such as modulation, amplification, and substitution, which enable describers to mediate between visual input and the communicative needs of visually impaired audiences (Bardini, 2020).

### 2.3 Underexplored dimensions in audio description training

Despite the growing body of research on AD scripts and audience reception, the pedagogical process through which describers develop competence within structured training contexts remains underexplored. Much existing work adopts product- or user-oriented perspectives, focusing on script quality or viewing experience (e.g., Abu Tair et al., 2023; Caro, 2016; Walczak and Fryer, 2017). While these approaches have yielded valuable insights into effective AD output, they offer limited understanding of how such competence is acquired over time.

In translation pedagogy, increasing attention has been paid to learning processes, with translation competence increasingly conceptualized and investigated through empirical studies of translator training and classroom practice (Galán-Mañas and Hurtado Albir, 2015; Hurtado Albir, 2017; Beeby et al., 2005; Tomozeiu et al., 2016). Applying process-oriented and competence-based approaches to AD can illuminate how students learn to integrate verbal description into complex audiovisual narratives, manage temporal and aesthetic constraints, and make interpretive decisions. However, longitudinal empirical studies tracing the development of AD competence remain limited, as existing research has mainly focused on product analysis and reception studies (e.g., Caro, 2016; Matamala and Remael, 2015), particularly in Chinese contexts where AD research is still emerging (Yan and Luo, 2019; Tor-Carroggio and Vercauteren, 2020).

Addressing this gap is essential for advancing AD pedagogy. A process-oriented investigation can reveal learners’ difficulties, clarify mechanisms of competence development, and inform evidence-based instructional design that supports the professionalization of future audio describers.

## 3 Research design

### 3.1 Research questions

Building on the identified lack of process-oriented research in AD training, this study investigates how student audio describers develop key competencies during classroom instruction and project-based practice in a Chinese university setting. Employing a mixed-methods approach that integrates quantitative analysis of task performance with qualitative analysis of textual data, classroom observation, and student reflection reports, the study traces the development of translation competence across the *what* and *how* dimensions throughout the learning process. Specifically, the study addresses the following research questions:

1. What developmental patterns emerge in student audio describers’ content selection competence throughout the course phases?
2. How do student audio describers progress in their application of different types of translation strategies during AD training?

### 3.2 Participants

The study recruited 30 undergraduate students from a foreign language university in Northeast China. The participants consisted of both male and female students, ranging from first- to third-year undergraduates, all majoring in Translation and Interpreting with English as the working language. Students admitted to this major are generally required to achieve English scores above 110 out of 150 in the National College Entrance Examination, indicating sufficient English reading and writing abilities for translation-related coursework. The participants enrolled in the AD course as an elective within the school. None had prior exposure to AD courses or systematic AD practice, making them suitable representatives of novice AD learners. With informed consent, the students participated in a one-term (approximately 16-week) AVT course comprising theoretical learning, skill training, and practical creation activities. As the evaluation criteria focused primarily on content selection and strategy application rather than linguistic accuracy, language proficiency was not the primary focus of assessment.

### 3.3 Procedure and materials

The course is a one-term program designed to develop students’ competence in screen AD, utilizing selected units from ADLAB PRO^1^ together with supplementary literature and professional guidelines. ADLAB PRO is an open-access modular training resource for AD, consisting of six modules divided into thematic units and providing customizable teaching materials such as videos, presentation slides, transcripts, tasks, and reading lists. Selected units from different modules were incorporated across multiple weeks of the course to support students’ learning and practice in Screen AD. As shown in Table 1, the course consists of weekly sessions, each lasting 80 min, with three in-class practice sessions spread throughout the term, occurring every 3 weeks (Weeks 4, 7, 10, 13 and 16).

**Table 1:** Course outline.

Week	Topic/content	ADLAB PRO modules and supplementary readings
Week 1	Course introduction	Watch original film
Week 2	Introduction to audio description: concept, history, and development trends	Module1-unit1, unit2
Week 3	Audio description workflow	Module1-unit5, Module2-unit2, unit3
Week 4	In-class practice	
Week 5	Screen AD: audio description and film narrative	Module2-unit1, literature from Kruger (2010), Mälzer-Semlinger (2012)
Week 6	Film techniques	*Pictures Painted in Words: ADLAB Audio Description Guidelines* (2.2) (Remael et al., 2015)
Week 7	In-class practice	
Week 8	Prioritizing information extraction	Module1-unit7, unit8, literature from Marzà Ibañez (2010), Wang et al. (2024)
Week 9	Language style and expression in audio description	Module2-unit4, unit5, unit6, unit7, unit8
Week 10	In-class practice	
Week 11	Translation techniques for audio description	Literature from Bardini (2020)
Week 12	Feedback and presentation	
Week 13	In-class practice	
Week 14	Evaluation standards for audio description	IO1 report, literature from Yan and Luo (2023)
Week 15	Feedback and presentation	
Week 16	In-class practice	

Throughout the course, students completed a series of practical AD tasks. They used CapCut to synchronize self-written AD scripts with film clips and submitted the final AD videos, scripts, and questionnaires at each phase. At the end of each phase, students completed a feedback questionnaire comprising five Likert-scale items (ranging from 1 = completely disagree to 5 = completely agree) and open-ended questions to capture both quantitative and qualitative perceptions of the tasks. The questionnaires differed across phases and were designed according to the specific learning objectives and task characteristics of each stage. Sample questionnaires are provided in Appendix A. Following the completion of all five phases, focus group interviews were conducted with students grouped by performance level to further explore their learning experiences (see Appendix B). The course was delivered by the same instructor throughout, with continuous assessment enabling the tracking of students’ competence development over time.

The course was delivered in cyclical phases, each consisting of 2 weeks of instruction followed by 1 week of in-class practice. Given the importance of contextual understanding for AD quality (Fryer, 2020), students viewed the complete film prior to completing task-based assignments.

Training tasks were based on Disney’s 1951 animated film *Alice in Wonderland*. Five excerpts were selected as practice materials, following the film’s chronological order and increasing progressively in length from 5 to 8 min (see Table 2). The excerpts were chosen as self-contained narrative segments to ensure comparability across tasks while allowing for the observation of developmental changes over time.

**Table 2:** Information of 5 excerpts from Alice in wonderland.

Excerpt	Duration	Brief title	Brief plots
1	5 m	The rabbit hole and pool of tears	Alice falls down the rabbit hole, lands in a hall of doors, cries a “Pool of Tears,” shrinks, and is swept away by the flood she creates.
2	5 m 13 s	The walrus and the carpenter	Alice encounters Tweedledee and Tweedledum, who recite the nonsensical poem The Walrus and the Carpenter instead of giving directions.
3	5 m 56 s	Chaos at the white rabbit’s house	At the White Rabbit’s house, Alice grows too large and becomes trapped, then shrinks again and escapes after a series of chaotic rescue attempts.
4	8 m 10s	The Mad Tea Party	The Mad Tea Party, where Alice joins the Mad Hatter, the March Hare, and the Dormouse in a chaotic “Unbirthday” celebration marked by illogical dialogue and constant seat switching.
5	8 m 27 s	The queen’s croquet game	Alice meets the queen of hearts after discovering card soldiers painting roses red and is forced into a chaotic croquet game using flamingos and hedgehogs.

Disney’s official AD, obtained from Disney+, was used as the professional benchmark for performance evaluation. As an industry-standard reference, it provides a normative basis for assessing students’ competence in content selection and translation strategy application in terms of temporal coordination, information prioritization, and narrative coherence.

### 3.4 Data collection and evaluation framework

Data were collected through a structured, process-oriented workflow across five stages of the course. The professional standard was first obtained by transcribing the official AD track from the selected film via Disney+, with the transcription manually verified to ensure lexical accuracy and faithful representation of translation strategies. Learner data were gathered continuously throughout the term via the Chaoxing online learning platform, which served as the central repository for all student outputs, including AD scripts, CapCut-produced soundtrack videos, Likert-scale ratings, and feedback questionnaire responses. Semi-structured focus group interviews were conducted at the end of the course to obtain qualitative perspectives on learners’ experiences and perceived development.

Following data collection, a mixed-methods approach was adopted for analysis. All quantitative data, comprising content selection scores, translation strategy ratings, and questionnaire and Likert-scale results, were first consolidated in Microsoft Excel before being imported into SPSS. SPSS was used to identify performance trends, conduct statistical comparisons, and generate visualizations illustrating learner progress across the five stages. For qualitative data, audio recordings of the focus group interviews were transcribed using Netease Workspace and subsequently coded and analyzed in ATLAS.ti to surface recurring themes relating to learners’ translation strategies and reflective processes.

Given the time-constrained and function-oriented nature of screen AD (Fryer, 2016), professionally produced AD scripts were used as reference benchmarks for performance evaluation. To preserve the authenticity of student output, the benchmark scripts were not disclosed prior to task completion and were shared with learners only after each stage for reflective comparison. Students were, however, familiarised with AD evaluation criteria and relevant theoretical frameworks through course readings and instruction, as outlined in the preceding section. Setton and Dawrant (2016, p.435) suggest the use of benchmark performances in interpreter training, recommending that high-quality expert samples be used to construct operationalizable assessment criteria and to calibrate evaluator judgement. Similarly, Han (2020) confirms the validity of comparative evaluation in contexts where performance is shaped by situational and communicative constraints. The task is intralinguistic in nature, as students were required to produce English AD scripts for an English-language film, transposing visual information into spoken English description without cross-linguistic transfer.

As noted earlier, AD involves the intersemiotic transposition of visual information into verbal description; accordingly, students’ translation strategy competence was assessed using Bardini’s (2020) framework, which conceptualizes such descriptive techniques within a translational paradigm. Of the 14 strategies proposed, nine were identified in the *Alice in Wonderland* AD and further grouped into basic, content-handling, and professional strategies, reflecting increasing levels of technical and cognitive demand (see Table 3).

**Table 3:** Three types of strategies based and categorized on Bardini’s (2020) AD techniques.

Type	Technique	Definition
Basic strategy	Iconic description	Denotative AD of a fragment of the audiovisual source text.
	Integral iconic description	Describing general-knowledge elements or body language literally, rather than using conventional labels or interpretive meaning.
Content-handling strategy	Reduction	Very brief (or inexistent) AD
	Generalization	Overly general or superficial description where greater detail or precision is feasible within the silent gap.
	Compensation	Non-simultaneous description, occurring before or after the corresponding visual event.
Professional strategy	Substitution	Interpretation of extra-linguistic elements, such as gestures or facial expressions.
	Modulation	Shifted descriptive focus compared to the original audiovisual text.
	Amplification	Expanded AD, adding information beyond the audiovisual text through techniques such as addition, explanation, or explication.
	Technical description	Using technical terminology related to the nature of the audiovisual material.

In each phase, 12 scenes were selected to examine students’ competence. For content selection, a 4-point scale was used: 0 = no description of the benchmarked scene; 1 = description with major deviations from the professional version; 2 = partial alignment, with minor prioritization differences; 3 = near-complete adherence to professional standards.

Evaluation focused on content selection and translation strategy application, specifically on whether students’ AD scripts identified and described the same key visual elements and scenes as the professional benchmark. Linguistic features such as word choice, grammatical accuracy were not included as assessment criteria, as the evaluation was concerned with content-level decision-making.

## 4 Results

Thirty students initially participated in the study. Due to incomplete submissions across the five phases, data from 26 students were retained for analysis. To examine competence development over time, students were ranked according to their cumulative content selection scores and assigned serial numbers from No. 1 (highest) to No. 26 (lowest). Based on these rankings, participants were further grouped into high-achieving (No. 1–8), medium-achieving (No. 9–17), and low-achieving (No. 18–26) groups to facilitate comparison of developmental trends across proficiency levels.

### 4.1 Content selection development

This section examines how students’ content selection competence evolved across the five phases of the course. Content selection refers to the process by which students identify and prioritize visually salient and narratively significant elements for verbalization. To capture both overall trends and individual variation, scores were analyzed both overall and by performance level.

**Figure 1:**
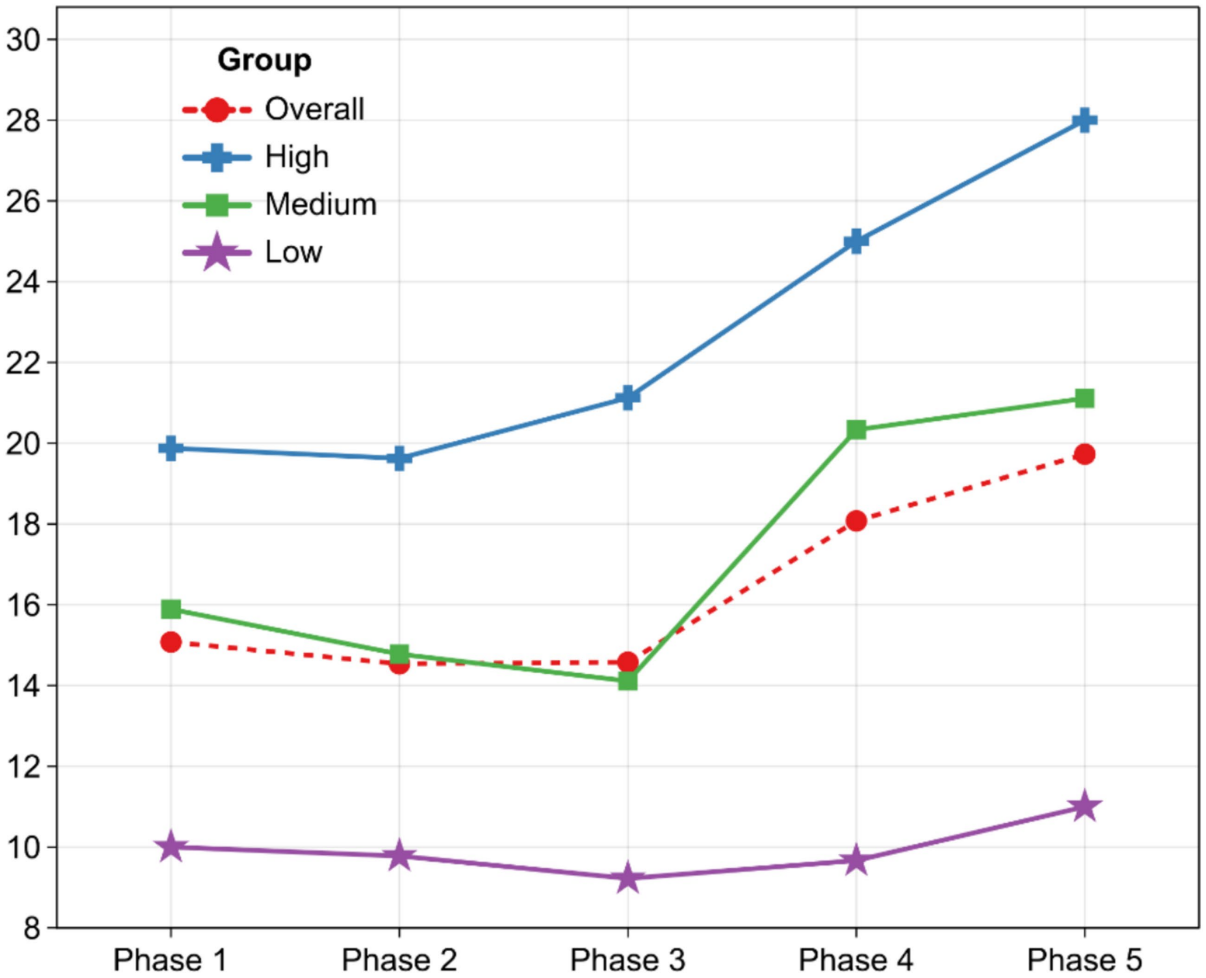
Line chart showing the average content selection scores for the overall group and for each performance level.

Figure 1 illustrates the content selection performance of students across five phases, categorized by achievement level. Overall, students demonstrated steady progress, with average scores showing a notable rise from Phase 3 to Phase 5.

The high-achieving group maintained consistently strong performance, with only a minor dip in Phase 2 and a continuous upward trend thereafter, reaching 28 points in the final phase. The medium group exhibited a more fluctuating pattern, with scores dipping in Phases 2 and 3 before rebounding sharply in Phase 4, eventually aligning with the overall trend.

In contrast, the low-achieving group showed minimal variation, with scores remaining around 10 points throughout. Although a slight improvement was observed in the final phase, their contribution to the overall increase was marginal.

Notably, the rise in the overall average was primarily driven by the substantial progress made by the high- and medium-achieving students. This divergence highlights widening performance gaps across groups and indicates that improvement was unevenly distributed.

Table 4 presents a statistical overview of students’ content selection scores across five phases. In the first three phases, mean and median scores remained relatively low and stable, with means around 15 and medians between 15 and 16. The slight dip in the mean from Phase 1 (15.077) to Phase 2 (14.538), followed by a minimal rebound in Phase 3 (14.577), indicates little substantive change in early performance. These fluctuations were not statistically significant, as reflected in high *p* values (*p* = 0.648 and 0.709) and negligible effect sizes (Cohen’s *d* = 0.091 and 0.074).

**Table 4:** Statistical analysis of content selection scores.

Phase	Mean	Median	SD	Significance (vs Phase 1)		
1	15.08	16	5.89	*t*	*p*	Cohen’s *d*
2	14.54	15	5.60	0.462	0.648	0.091
3	14.58	16	6.69	0.377	0.709	0.074
4	18.08	22	7.48	−2.133	0.043*	0.418
5	19.73	23.5	9.13	−2.805	0.010**	0.550

^*^p < 0.05, ^**^p < 0.01.

A clear improvement emerged in Phase 4, where the mean increased to 18.077 and the median rose sharply to 22, marking the first statistically significant difference from Phase 1 (*p* = 0.043, Cohen’s *d* = 0.418). This upward trend continued in Phase 5, with further increases in both mean (19.731) and median (23.5), alongside a stronger effect size (*p* = 0.010, Cohen’s *d* = 0.550).

Notably, median scores increased more substantially than mean scores in later phases, suggesting that more than half of the students achieved performance above the group average. At the same time, score variability increased, with standard deviation rising from 5.886 in Phase 1 to 9.128 in Phase 5. This pattern indicates widening performance gaps, as some students improved markedly while others continued to struggle.

### 4.2 Strategy application development

This section examines how students’ application of translation strategies developed across the five phases. Translation strategy application concerns the how dimension of audio description, namely the linguistic and technical decisions through which selected visual content is rendered into coherent verbal output.

**Figure 2:**
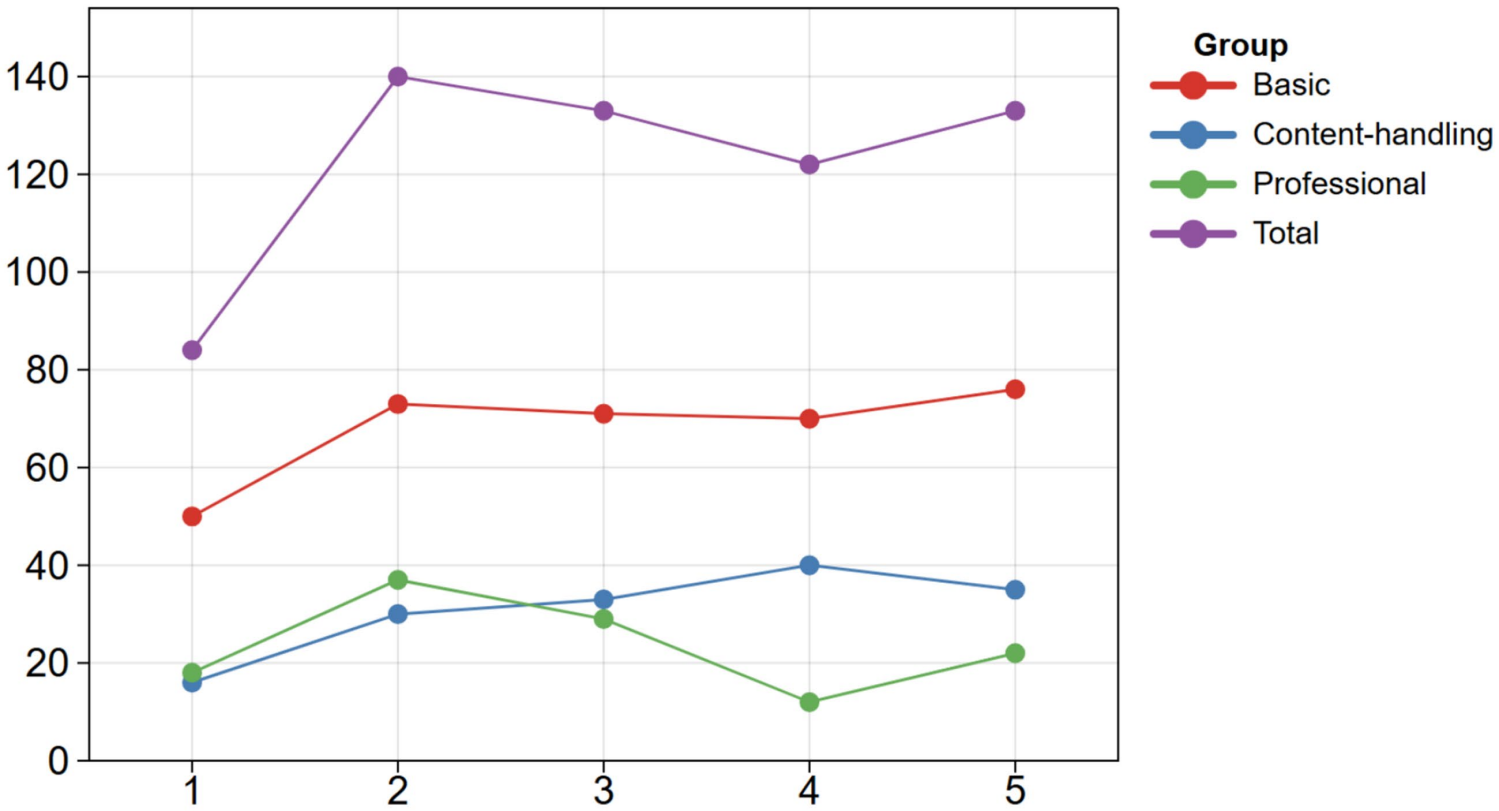
Students’ correct application of translation strategies of 3 types across 5 phases.

Figure 2 illustrates the trajectory of translation strategy application across five phases, disaggregated by strategy type. A pronounced surge in total translation strategy employment occurs between Phase 1 and Phase 2, signaling an initial breakthrough in students’ engagement. Subsequent phases (Phases 2–5) show no statistically significant differences, indicating a stabilization plateau following the early gains.

Basic strategy utilization mirrors this overall trajectory, exhibiting an early spike followed by sustained equilibrium. This pattern suggests that once foundational strategic competencies are acquired, students maintain relatively consistent application.

Content-handling strategies demonstrate a more gradual ascent, with sustained elevation from Phase 2 onwards relative to baseline performance. This incremental progression reflects students’ evolving capacity for content manipulation and restructuring, which develops more slowly than basic descriptive skills.

Professional strategies exhibit marked volatility, with erratic fluctuations rather than linear progression. The absence of clear developmental momentum—evidenced by Phase 4 performance falling below Phase 1 levels—underscores the persistent challenges inherent in mastering advanced strategic competencies.

Cross-categorical analysis reveals a clear hierarchy in strategy deployment, with basic strategies dominating usage patterns across all phases, while professional strategies remain consistently underutilized, reflecting their complexity and the extended timeframe required for mastery.

**Figure 3:**
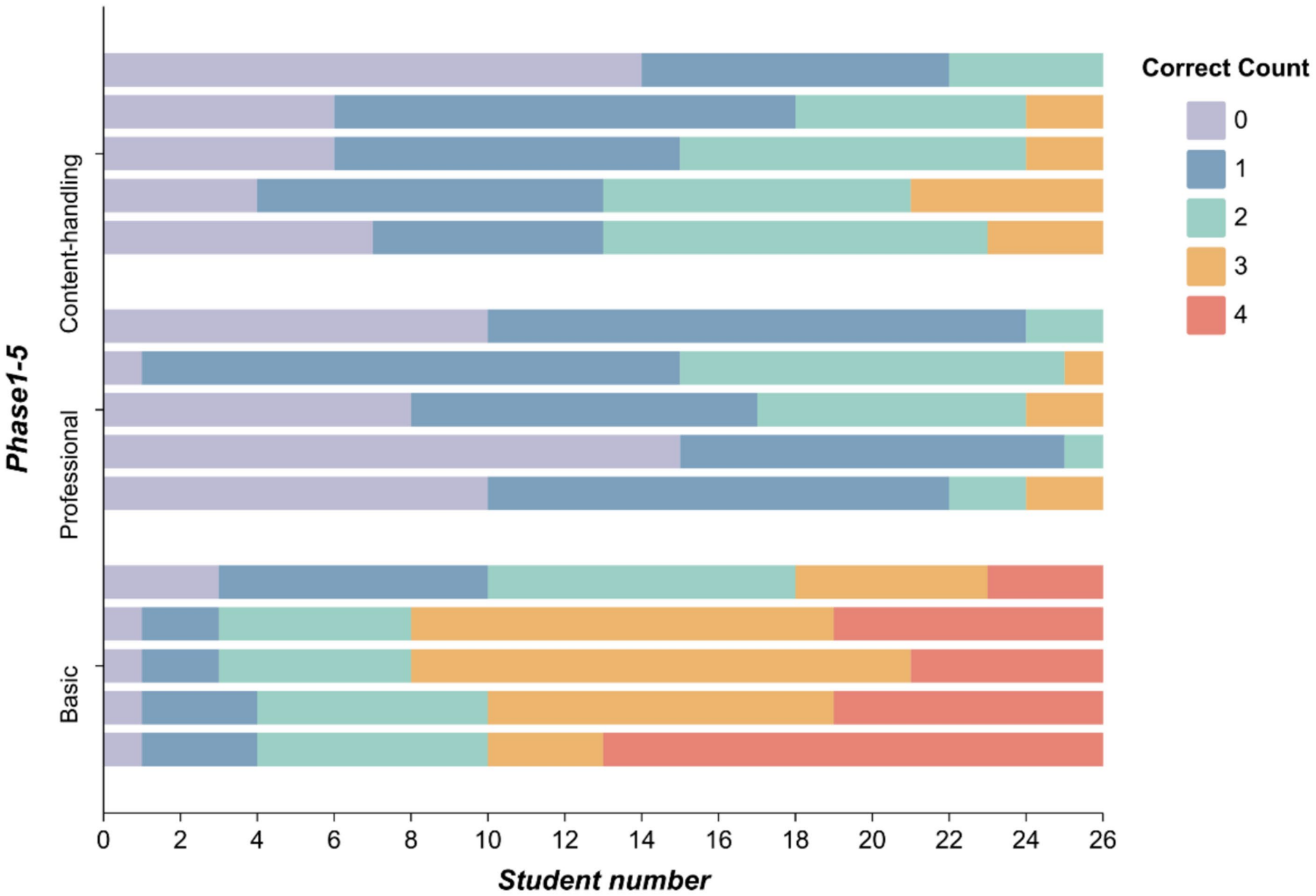
Proportion of students correctly applying translation strategies of three types.

Figure 3 shows the proportion of students who correctly applied basic, professional, and content-handling strategies across Phases 1 to 5, based on the number of correctly applied strategies (0–4). Among the three categories, basic strategies show the most substantial improvement over time. From Phase 1 to Phase 5, the proportion of students applying all four basic strategies increased markedly, while the share of students applying zero or only one basic strategy declined, indicating overall progress in foundational strategic competence.

For content-handling strategies, the overall trend was positive. The number of students correctly applying two or three strategies gradually increased, while those applying zero or one strategy decreased. However, no student correctly applied all four content-handling strategies in any phase, and only a small number achieved three correct applications, indicating that improvement did not translate into full mastery.

In contrast, professional strategies showed no clear upward trend. Although the proportion of students with zero or one correct application declined in Phase 2, this improvement was not sustained, and by Phase 5 it returned to a level comparable to Phase 1. As with content-handling strategies, no student achieved full marks in any phase, and only a few applied three strategies correctly. This irregular pattern suggests that professional-level strategies consistently posed greater challenges throughout the training.

## 5 Discussion

### 5.1 Content selection

Overall, students’ content selection competence showed a clear upward trajectory, with a particularly marked improvement after Phase 4, indicating strong late-stage momentum. At the same time, individual differences became increasingly salient as training progressed. Score distributions revealed diverging developmental patterns, characterized by elite advancement, mid-level fluctuation, and relative stability among low-performing students.

In the early phases, students were newly exposed to AD and relied largely on teacher instruction and limited examples. Their performance reflected a rudimentary understanding of AD as surface-level visual description, resulting in generally low and narrowly distributed scores. At this stage, performance differences were mainly shaped by individual learning attitudes and engagement, as reflected in classroom participation and task completion.

Substantial qualitative improvement emerged in Phases 4 and 5, particularly among high- and mid-performing students. This shift coincided with a deeper understanding of AD principles and a move toward a more target-oriented approach, consistent with findings that internal sub-competences and audience orientation contribute significantly to translation quality (Beeby et al., 2005; Nord, 1997). Questionnaire data indicated that students’ perceptions evolved from vague notions of description to more nuanced views emphasizing narrative coherence and audience experience. Early tendencies toward verbose but poorly aligned descriptions gradually diminished, while a listener-centered orientation became more evident, echoing the importance of audience awareness emphasized in previous research (ADLAB PRO IO2, 2017; Mendoza and Matamala, 2019; Yan and Luo, 2024).

Interviews with high-achieving students further revealed a qualitative shift in focus from describing isolated visuals to managing narrative flow and auditory space. Some students independently recognized the need to preserve key sound effects—an aspect not explicitly taught but noted in earlier studies (Hutchinson and Eardley, 2023; Lopez et al., 2021):

Student 1: “I realized that visually impaired audiences rely heavily on sound. Important effects, such as sighs or laughter, should be left unobstructed. If we describe over them, the description competes with the original audio and disrupts the listening experience.”

**Figure 4:**
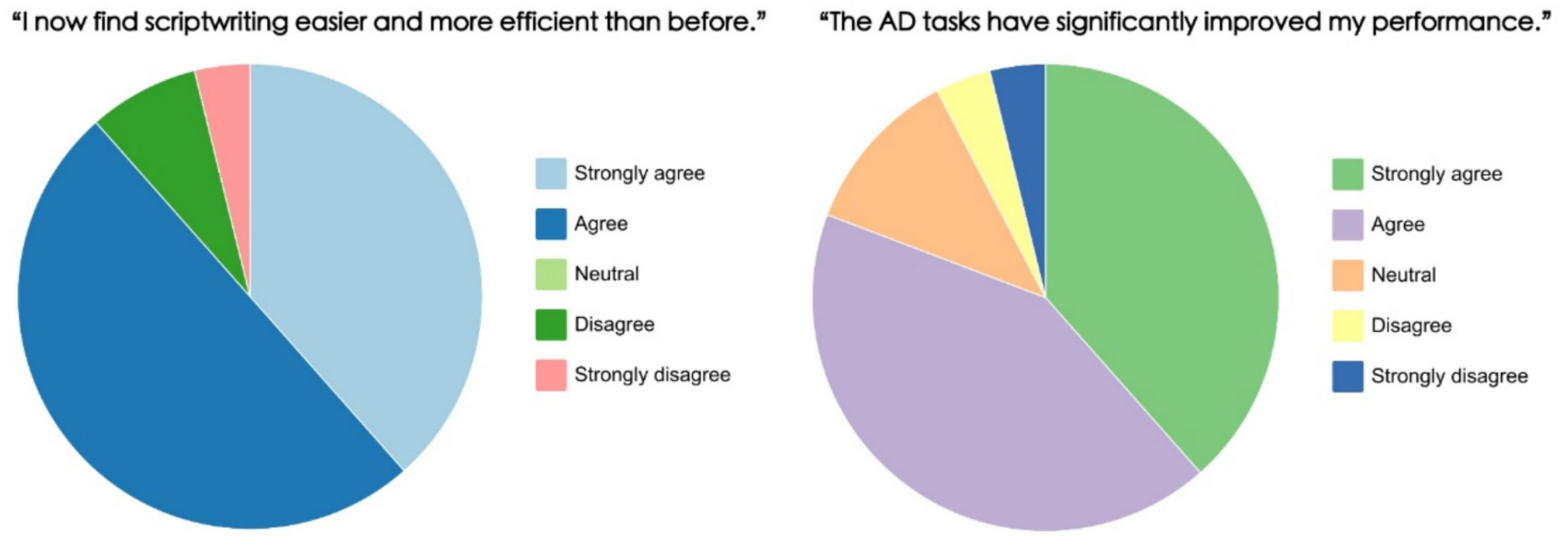
Students’ feedback from Phase 4 and 5.

Finally, accumulated practice and exposure to official AD contributed to students’ development. Questionnaire responses from Phases 4 and 5 suggest that most students perceived AD tasks as increasingly effective and scriptwriting as more efficient (see Figure 4). High-achieving students reported that comparison with professional AD supported information filtering in earlier stages and refined language expression in later stages.

A clear shift was also observed in students’ perceived challenges over time. Beginner students primarily struggled with deciding what to describe, whereas more advanced learners increasingly identified precise language use as the main difficulty. This shift further reflects the development of content selection competence:

Student 2: “As I progressed to the later stages, deciding what to describe became much easier, almost subconscious. The real challenge was how to phrase it. When I compared my script with the official version, I realized how much my wording and vocabulary still needed improvement.”

By contrast, low-achieving students showed limited progress across phases. Their scripts often remained incomplete or overly brief, with some scenes minimally described or omitted altogether. As the course was elective, inconsistent effort may have contributed to this pattern, highlighting the role of psychological competence in translation learning, which is often underexplored in classroom-based research (Wu et al., 2019).

Pedagogically, these findings point to two implications. First, the largely linear improvement observed among most students suggests that content selection competence benefits from explicit instruction, clear criteria, and iterative feedback, including benchmark comparison. Second, the diverging developmental trajectories and the persistent low performance of one group indicate the need for differentiated instruction: while high-achieving students rapidly shift their focus to linguistic refinement (the *how*), low-achieving students require continued support in identifying salient visual information (the *what*).

### 5.2 Translation strategy application

Compared with content selection, the application of translation strategies showed greater fluctuation across phases and among students, particularly with regard to advanced strategies. While content selection concerns what to describe, strategy application determines how visual information is rendered linguistically. Basic and content-handling strategies are closely aligned with content selection, as both rely on identifying and verbalizing essential narrative elements. In contrast, professional strategies depend more heavily on advanced competences, including linguistic expressiveness and cinematic awareness. As a result, the development of content selection and strategy application does not always proceed in parallel.

Interview data confirmed this asymmetry. High-achieving students reported that they rarely applied strategies deliberately, a finding consistent with the Dreyfus and Dreyfus (1986) novice–expert model, which characterizes expert performance as largely intuitive. Interestingly, some low-achieving students also demonstrated occasional appropriate strategy application, suggesting that strategies may become partially internalized regardless of overall performance level. The sharp improvement observed between the first two phases likely reflects the initial awareness-raising effect of instruction. Subsequently, strategy application became less consciously monitored and more variable, producing the non-linear developmental patterns observed—patterns that contrast with the steady progress found in content selection.

The three strategy dimensions, basic, content-handling, and professional, followed uneven developmental trajectories. Basic strategies were the most frequently used and showed the clearest improvement, particularly after students were exposed to the official AD script, which enhanced their understanding of prioritization. Content-handling strategies developed more gradually, as they required growing narrative awareness and improved temporal coherence. Professional strategies, however, remained consistently weak across groups. These strategies demand refined language use and sophisticated narrative and cinematic interpretation, corresponding to describers’ competence in cinematographic language and image semiotics (Matamala and Orero, 2007). Although concepts such as cinematic AD and Auteur Description have been shown to enhance accessibility for visually impaired audiences (Fryer and Freeman, 2012; Szarkowska, 2013; Thompson, 2018), such expertise typically develops over a longer period than a single semester, explaining students’ limited performance in this dimension (see Table 5).

**Table 5:** Examples of professional strategies with a low number of correct applications by students.

Official AD	Strategy	Phase	Students applying correctly (*n*)
The March Hare looks at us.	Technical Description	4	0
Peeking from behind a high-backed red chair, Alice spies colorful teapots tooting steam into the air.	Substitution	4	8
In a mad dash, the playing cards arched their backs, forming hoop shaped wickets for the rolling hedgehog to pass under.	Amplification	5	6

The film screenshot is used solely for academic analysis purposes. Source: Geronimi et al. (1951), dir. Clyde Geronimi, Wilfred Jackson, and Hamilton Luske. © Walt Disney Productions.

Overall, the findings reveal distinct developmental trajectories for content selection and strategy application. Content selection improved steadily and linearly, reflecting students’ increasing awareness of narrative structure and audience needs. Translation strategy application, by contrast, followed a more fluctuating and non-linear path, indicating its reliance on higher-level linguistic, cognitive, and narrative competences. Pedagogically, this suggests the need for a tiered instructional design that prioritizes foundational descriptive skills before progressively integrating advanced narrative and stylistic strategies.

In terms of strategy instruction specifically, the largely intuitive and unstable nature of strategy application indicates that presenting theoretical frameworks alone is insufficient for mastery. Instead, reflective practice should be emphasized. For example, students may first draft scripts intuitively and then systematically compare them with professional benchmarks to identify and articulate the strategies employed. Moreover, the persistent difficulty with professional strategies highlights the need for sustained, long-term training that extends beyond the scope of an introductory AD course and points to the value of incorporating interdisciplinary content, including cinematographic language and image semiotics, into AD curricula.

## 6 Conclusion

This study examined the development of students’ competence in content selection and translation strategy application in an AVT course. Drawing on performance data, questionnaires, and interviews, the findings reveal divergent developmental patterns across the two dimensions. Content selection showed steady improvement across phases, reflecting students’ increasing awareness of AD’s narrative function and audience needs. By contrast, strategy application was more variable, particularly with respect to advanced strategies. While basic and content-handling strategies largely developed in parallel with content selection, professional strategies lagged behind, as they require more advanced linguistic and narrative competence.

Several limitations should be acknowledged. The relatively small sample size of 26 students from a single institution limits the generalizability of the findings. In addition, the use of a single animated film as training material restricts insights into genre-specific variation in AD competence. Finally, the one-semester training period may not have been sufficient for the full development of advanced strategies.

Future research could extend this line of inquiry by including participants from multiple institutions and educational backgrounds. Expanding the range of AD materials across genres would further illuminate how students adapt descriptive and strategic skills in different contexts. Longer-term training designs are also needed to capture the sustained development of advanced strategy application over time.

## Data Availability

The raw data supporting the conclusions of this article will be made available by the authors, without undue reservation.

## Footnotes

1
https://www.adlabpro.eu/coursematerials/
