# Supplementary material for: Learning what and how to describe: longitudinal development of content selection and strategy application in audio description training
Source: Front Psychol. 2026 Jul 1;17:1771684. doi: 10.3389/fpsyg.2026.1771684 (PMC13371935; doi:10.3389/fpsyg.2026.1771684)
Supplement: Supplementary file 1 [file Data_Sheet_1.docx]

**APPENDIX A Feedback questionnaire**

Likert-scale items

| Phase | Questions | Completely disagree | Disagree | Neutral | Agree | Completely agree |
| --- | --- | --- | --- | --- | --- | --- |
| 1 | I have clearly understood the core functions of AD and the needs of its target audience. | 1 | 2 | 3 | 4 | 5 |
|  | I can distinguish AD scripts from other forms of translation. | 1 | 2 | 3 | 4 | 5 |
|  | In simple scenes, I can accurately identify the key visual elements that should be described. | 1 | 2 | 3 | 4 | 5 |
|  | I have gained a preliminary understanding of the AD workflow. | 1 | 2 | 3 | 4 | 5 |
|  | In classroom practice, I can initially balance descriptive completeness with linguistic conciseness. | 1 | 2 | 3 | 4 | 5 |
| 2 | In simple scenes, I can accurately identify the key visual elements that should be described. | 1 | 2 | 3 | 4 | 5 |
|  | In classroom practice, I can initially balance descriptive completeness with linguistic conciseness. | 1 | 2 | 3 | 4 | 5 |
|  | In dynamic scenes, I can effectively capture key action information. | 1 | 2 | 3 | 4 | 5 |
|  | In complex scenes, I can quickly determine which visual information should be prioritized for description. | 1 | 2 | 3 | 4 | 5 |
|  | I can prioritize core narrative elements according to time constraints. | 1 | 2 | 3 | 4 | 5 |
| 3 | In complex scenes, my prioritization of information is supported by reasonable justification. | 1 | 2 | 3 | 4 | 5 |
|  | I can prioritize core narrative elements according to time constraints. | 1 | 2 | 3 | 4 | 5 |
|  | In complex scenes, I can quickly determine which visual information should be prioritized for description. | 1 | 2 | 3 | 4 | 5 |
|  | I can use appropriate language styles and precise wording according to the atmosphere of a scene. | 1 | 2 | 3 | 4 | 5 |
|  | I have initially mastered translation strategies used in AD. | 1 | 2 | 3 | 4 | 5 |
| 4 | Explanations of AD translation techniques in class have directly helped me complete practical tasks. | 1 | 2 | 3 | 4 | 5 |
|  | During pauses between dense dialogues, I can efficiently insert descriptions without interfering with the original soundtrack. | 1 | 2 | 3 | 4 | 5 |
|  | I have an accurate understanding of the key points involved in integrating narration when writing AD scripts. | 1 | 2 | 3 | 4 | 5 |
|  | I can proficiently apply at least three translation techniques to optimize AD scripts. | 1 | 2 | 3 | 4 | 5 |
|  | Compared with before, I now feel more comfortable and efficient when writing AD scripts. | 1 | 2 | 3 | 4 | 5 |
| 5 | I can optimize AD scripts according to assessment criteria such as information completeness and language fluency. | 1 | 2 | 3 | 4 | 5 |
|  | In the final practice task, I can independently produce AD works that meet industry standards. | 1 | 2 | 3 | 4 | 5 |
|  | I can evaluate the quality of AD scripts. | 1 | 2 | 3 | 4 | 5 |
|  | The multiple practical tasks in the course have significantly improved my content selection and translation skills. | 1 | 2 | 3 | 4 | 5 |
|  | I am interested in engaging in AD-related work in the future. | 1 | 2 | 3 | 4 | 5 |

Open-ended Questions

**Phase 1**

1. What was the greatest challenge you encountered when attempting AD scriptwriting for the first time? How did you deal with it?

**Phase 2**

1. Describe an experience in which you adjusted your content selection due to time pressure.
2. In describing dynamic scenes, which visual elements did you find the most difficult to capture, and why?

**Phase 3**

1. In this practical task, how did you determine which visual elements should be selected for description in complex scenes?
2. In your opinion, what should the extraction of visual information in AD be closely related to?

**Phase 4**

1. Do you think the difficulty level changed across the four tasks?
2. Did your personal condition or state affect your task performance? If so, how?

**Phase 5**

1. Comparing your AD scripts from Phase 1 and Phase 5, in what aspects do you think you made the greatest progress?
2. If you were to take this course again, which aspects would you like to spend more time on?

# APPENDIX B Focus group interview outline

1. Could you walk us through your complete workflow when completing an AD task?
2. When applying the compensation strategy (i.e., describing content earlier or later than it appears on screen), how do you make that decision?
3. When faced with a complex scene, how do you decide what to describe and what to leave out? What is your primary criterion for selecting information?
4. To what extent did you refer to the official AD script? In what specific ways was it most helpful to you?
5. When producing the AD for Alice in Wonderland, did you look up additional materials to understand the plot or symbolic meanings? If so, how did this influence your script writing?
6. How helpful did you find the theoretical content of the course for your actual AD production? Which part gave you the most inspiration?
7. Reflecting on the semester’s practice as a whole, which aspect of AD production do you feel you have improved the most?
8. Conversely, which aspects do you still find most challenging, or feel you most need to work on?
9. Overall, which stage of the AD production process do you find easiest, and which presents the greatest challenge?
10. Through this semester’s AD practice, how has your understanding of how visually impaired audiences experience audiovisual content changed?
